# Complement Activation Profile in Myasthenia Gravis Patients: Perspectives for Tailoring Anti-Complement Therapy

**DOI:** 10.3390/biomedicines10061360

**Published:** 2022-06-09

**Authors:** Nicola Iacomino, Fiammetta Vanoli, Rita Frangiamore, Marta Ballardini, Letizia Scandiffio, Federica Bortone, Francesca Andreetta, Fulvio Baggi, Pia Bernasconi, Carlo Antozzi, Paola Cavalcante, Renato Mantegazza

**Affiliations:** Neurology IV–Neuroimmunology and Neuromuscular Diseases Unit, Fondazione IRCCS Istituto Neurologico Carlo Besta, 20133 Milan, Italy; nicola.iacomino@istituto-besta.it (N.I.); fiammetta.vanoli@istituto-besta.it (F.V.); rita.frangiamore@istituto-besta.it (R.F.); marta.ballardini@istituto-besta.it (M.B.); letizia.scandiffio@istituto-besta.it (L.S.); federica.bortone@istituto-besta.it (F.B.); francesca.andreetta@istituto-besta.it (F.A.); fulvio.baggi@istituto-besta.it (F.B.); pia.bernasconi@istituto-besta.it (P.B.); carlo.antozzi@istituto-besta.it (C.A.); renato.mantegazza@istituto-besta.it (R.M.)

**Keywords:** myasthenia gravis, autoimmunity, biomarkers, complement system, anti-complement therapy

## Abstract

The complement system plays a key role in myasthenia gravis (MG). Anti-complement drugs are emerging as effective therapies to treat anti-acetylcholine receptor (AChR) antibody-positive MG patients, though their usage is still limited by the high costs. Here, we searched for plasma complement proteins as indicators of complement activation status in AChR-MG patients, and potential biomarkers for tailoring anti-complement therapy in MG. Plasma was collected from AChR-MG and MuSK-MG patients, and healthy controls. Multiplex immunoassays and ELISA were used to quantify a panel of complement components (C1Q, C2, C3, C4, C5, Factor B, Factor H, MBL, and properdin) and activation products (C4b, C3b, C5a, and C5b-9), of classical, alternative and lectin pathways. C2 and C5 levels were significantly reduced, and C3, C3b, and C5a increased, in plasma of AChR-MG, but not MuSK-MG, patients compared to controls. This protein profile was indicative of complement activation. We obtained sensitivity and specificity performance results suggesting plasma C2, C3, C3b, and C5 as biomarkers for AChR-MG. Our findings reveal a plasma complement “C2, C3, C5, C3b, and C5a” profile associated with AChR-MG to be further investigated as a biomarker of complement activation status in AChR-MG patients, opening new perspectives for tailoring of anti-complement therapies to improve the disease treatment.

## 1. Introduction

Myasthenia gravis (MG) is a prototypic antibody-mediated autoimmune disease of the neuromuscular junction (NMJ); its clinical hallmarks are weakness and fatigability of ocular, bulbar, and skeletal muscles. Most patients (80–85%) have antibodies (Abs) directed against the nicotinic acetylcholine receptor (AChR) on the post-synaptic membrane; other autoantigens are the muscle-specific kinase (MuSK) and the low-density lipoprotein receptor-related protein 4, both involved in AChR clustering at the NMJ [1,2]. Anti-AChR Abs belong to the complement-fixing IgG1 and IgG3 subclasses and their pathogenicity is mainly due to complement system activation, leading to membrane attack complex (MAC) formation and deposition on the NMJ, focal lysis of the post-synaptic membrane, disruption of post-junctional folds, and ultimately reduction in functional AChRs [3,4]. This occurs via the classical complement pathway with C1q binding to the Fc domain of the anti-AChR Ab and activation of the complement cascade; critical steps are cleavage of C3 into C3a and C3b, and of C5 in C5a and C5b, the latter leading to MAC (C5b-9) formation [5,6]. The role of the complement system has been largely demonstrated in MG patients and animal models [7,8,9,10]. Preclinical studies in experimental autoimmune myasthenia gravis (EAMG) have demonstrated the efficacy of complement inhibition via recombinant proteins, chemicals, monoclonal Abs, and small interfering RNA (siRNA) in improving MG symptoms [11,12,13,14,15]. Since complement activation is the result of a multi-step cascade of events, many potential targets are available as candidates for therapeutic intervention. Eculizumab, a humanized monoclonal Ab-binding C5, has recently been approved by the FDA, Japan PMDA, and EMA for the treatment of AChR-MG patients with generalized disease (gMG) [16,17,18]. Eculizumab inhibits C5 cleavage into C5a and C5b, hence blocking MAC formation. The drug resulted in the clinical benefit of refractory AChR-positive gMG patients in the phase III REGAIN trial, and the following open-label extension study, demonstrating that eculizumab is able to elicit a rapid and sustained improvement in muscle strength [19,20,21,22]. Zilucoplan, a small macrocyclic peptide that allosterically inhibits C5 cleavage, also generated meaningful improvement in patients with moderate to severe gMG in the phase III RAISE study (https://clinicaltrials.gov/ct2/show/NCT04115293; https://www.ucb.com/stories-media/Press-Releases/article/UCB-announces-positive-data-in-myasthenia-gravis-with-zilucoplan-phase-3-study-results, accessed on 8 April 2022), strengthening the evidence of complement inhibition efficacy in gMG [23].

Current MG treatments are based on anti-cholinesterase drugs, chronic immunosuppression with corticosteroids, immunomodulation, and thymectomy in selected patients [24]. However, about 10% of patients are treatment refractory or intolerant to immunosuppressive therapies over time; moreover, up to 80% of them do not reach complete stable remission [25]. Complement inhibitors are highly promising for the treatment of refractory MG, at the same time offering the opportunity to limit, and hopefully avoid, chronic immunosuppression with corticosteroids. Response to anti-complement therapies may vary depending on patient-specific factors, different complement activation degrees, or pathogenic mechanisms other than complement activation, such as antigenic modulation or AChR blockage [26]. Therefore, the identification of the greater or lesser susceptibility of patients to respond to such complement inhibitors may greatly facilitate their introduction in clinical practice. At present, complement-related biomarkers in MG are missing.

Herein, we aimed at identifying plasma complement components or activation products as possible indicators of the complement activation degree in AChR-MG patients. By performing a comprehensive plasma complement protein profiling, we found significant changes in C2, C3, C5, C3b, and C5a in AChR-MG patients compared to healthy controls, suggesting the potential value of these proteins as complement-related biomarkers for MG.

## 2. Results

### 2.1. Complement Component Profiling Shows C2 and C5 Decrease, and C3 Increase in Plasma of AChR-MG Patients

Complement components of the classical, alternative and lectin pathways were analyzed in plasma from AChR-MG patients, MuSK-MG patients, and healthy controls (Table 1). The following proteins were included: C1q, C2, C3, C4, C5, Factor B, Factor H, Mannose-Binding Lectin (MBL), and properdin. Bead-based multiplex immunoassays using the Luminex technology were used for all the complement cascade proteins except for C2 and Properdin, which were independently analyzed by ELISA.

**Figure 1:**
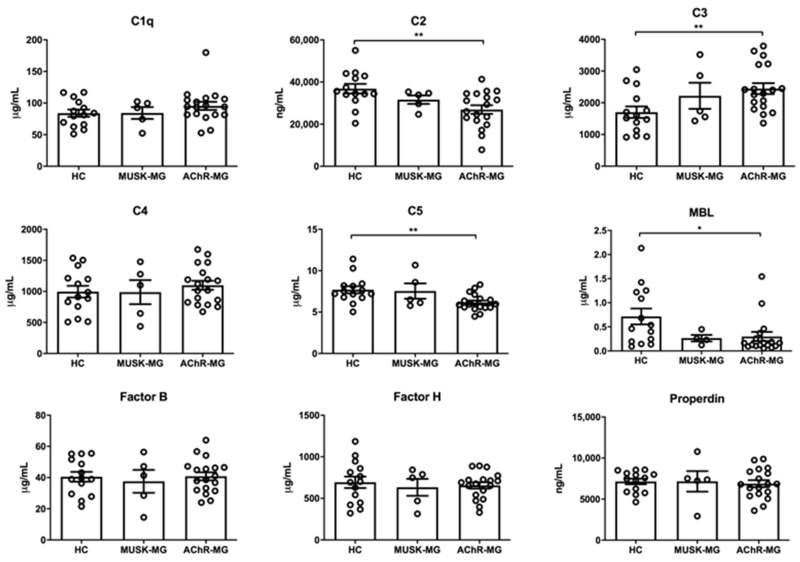
Quantification of complement protein components in plasma of healthy controls (HC), and MuSK-MG and AChR-MG patients. Protein concentrations of C1q, C3, C4, C5, MBL, Factor B, and Factor H were obtained by bead-based multiplex immunoassays on Luminex platform. C2 and properdin levels were estimated by a specific ELISA. For MBL data analysis, two outliers (one in the MuSK-MG and one in the AChR-MG group) were excluded based on the Grubbs’ test. No outliers were identified in the other protein datasets. A significant reduction in C2, C5 (** *p* < 0.01), and MBL (* *p* < 0.05) plasma levels was observed in AChR-MG patients compared to HC, accompanied by a significant increase in C3 (** *p* < 0.01). No difference in the concentration values of the remaining complement components was found among AChR-MG and MuSK-MG patients, and HC (*p* > 0.05). Data in the graphs correspond to mean concentration values ± standard error of the mean (SEM) obtained in each sample group. Mann–Whitney test, * *p* < 0.05; ** *p* < 0.01.

We found a significant decrease in plasma C2 and C5 levels in AChR-MG, but not in MuSK-MG, patients compared to healthy controls, suggesting complement consumption due to complement activation. On the contrary, plasma levels of C3, a key mediator of the complement cascade exerting direct effector functions in inflammation and innate immune activation [27], were abnormally increased in AChR-MG, but not in MuSK-MG patients compared to controls (Figure 1). Plasma levels of MBL, an important constituent of the innate immunity [28], were significantly lower in AChR-MG patients than those observed in healthy controls (Figure 1). Factor B and properdin, which are complement proteins specifically involved in the alternative pathway [29,30], showed similar concentrations among AChR-MG and MuSK-MG patients, and controls (Figure 1), suggesting that C2 and C5 consumption in AChR-MG patients may be mainly a result of activation of the classical pathway. The concentration of the other complement components was not different among the MG patients’ groups and controls (Figure 1). No difference in plasma concentration of C2, C3, C5, and MBL was observed between corticosteroid-naïve and -treated AChR-MG patients, and no correlation was found between the protein concentrations and the duration of IS treatment (data not shown), with the exception of MBL levels, that showed a trend to be higher in corticosteroid-naïve than corticosteroid-treated patients. C2, C3, C5, and MBL levels did not correlate either with disease severity evaluated by MG-specific Activities of Daily Living (MG-ADL) and MG Composite (MGC) scores or with anti-AChR Ab titers (data not shown). Based on clinical evaluation at the time of blood collection, AChR-MG patients were further classified as patients in pharmacological remission or symptomatic patients with gMG. No significant difference in the C2, C3, C5, and MBL levels was observed between the two groups of patients, in keeping with the lack of correlation between plasma concentrations of these proteins and MG severity (Supplementary Figure S1A).

### 2.2. Complement Activation Product Profiling Shows C5a and C3b Increase in Plasma of AChR-MG Patients

**Figure 2:**
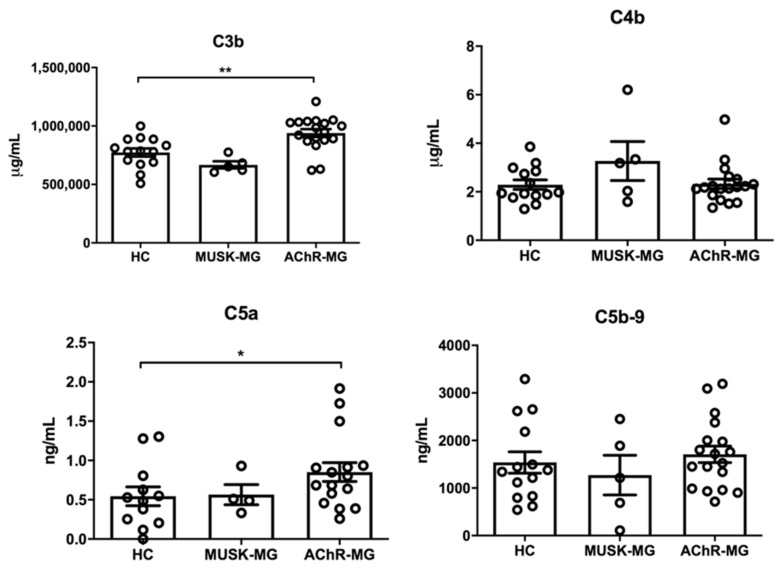
Quantification of complement activation products in plasma of healthy controls (HC), and MUSK-MG and AChR-MG patients. Protein concentrations of C3b and C4b were obtained by bead-based multiplex immunoassays on Luminex platform. C5a and C5b-9 levels were estimated by a specific ELISA. A significant increase in C3b (** *p* < 0.01) and C5a (* *p* < 0.05) levels was found in AChR-MG patients compared to HC. No difference in C4b and C5b-9 concentration was found among AChR-MG and MuSK-MG patients and HC (*p* > 0.05). Data in the graphs correspond to mean concentration values ± standard error of the mean (SEM) obtained in each sample group. Mann–Whitney test, * *p* < 0.05; ** *p* < 0.01.

C4b, C3b, C5a, and C5b-9 complement activation product levels were analyzed in plasma from AChR-MG, MuSK-MG, and healthy controls (Table 1). C4b and C3b were analyzed by bead-based multiplex immunoassays; C5a and C5b-9 levels were assessed separately by ELISA. We found a significant increase in plasma C3b and C5a levels in AChR-MG, but not in MuSK-MG, patients compared to healthy controls, suggesting complement activation (Figure 2). C3b levels positively correlated with those of C3 in AChR-MG patients (Supplementary Figure S1B). Plasma levels of C4b and C5b-9 were not significantly different in AChR-MG patients compared to MuSK-MG patients and controls (Figure 2).

No significant difference was observed in plasma concentration of the tested proteins between corticosteroid-naïve and -treated AChR-MG patients, and no correlation was found between the protein levels and the duration of IS treatment (data not shown). C3b and C5a concentration values did not correlate with MGC and MG-ADL scores in AChR-MG patients, or with anti-AChR Ab titers (data not shown). Accordingly, no significant difference in C3b and C5a levels was observed in AChR-MG patients in pharmacological remission compared to those with generalized symptoms (Supplementary Figure S1C).

### 2.3. Complement Activation Biomarkers in AChR-MG

**Figure 3:**
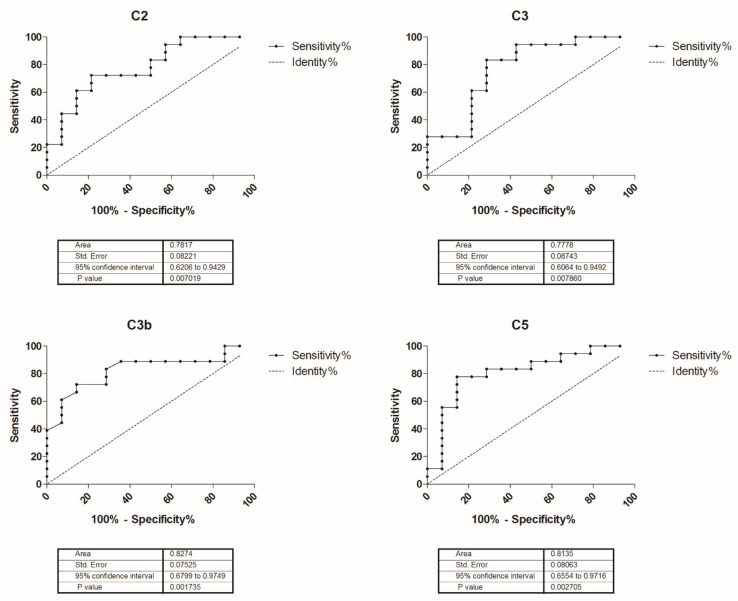
Potential value of C2, C3, C3b, and C5 as biomarkers for AChR-MG. Receiver operating characteristic (ROC) curves indicative of sensitivity and specificity of plasma C2, C3, C3b, and C5 as potential biomarkers able to discriminate AChR-MG and healthy controls. The true positive rate (sensitivity) on the *y*-axis is represented as a function of the false positive rate (100%-specificity%) on the *x*-axis. Higher y values correspond to a higher sensitivity, and lower x values correspond to a higher specificity.

Quantification of complement components and activation products in AChR-MG patients revealed a plasma protein profile expression of complement activation (Table 2). We assessed the potential value of the altered complement proteins C2, C3, C5, C3b, and C5a as biomarkers for AChR-MG. By Receiver operating characteristic (ROC) curve analysis, we obtained sensitivity and specificity performance of plasma C2, C3, C3b, and C5 as biomarkers able to discriminate between AChR-MG patients and healthy controls (Figure 3).

## 3. Discussion

Autoantibodies to the AChR are present in most MG patients, causing NMJ impairment ultimately leading to muscle weakness and fatigability [1]. Their pathogenicity is mainly due to complement activation and complement-induced destruction of the postsynaptic membrane, as demonstrated by studies in MG patients and animal models [7,8,11,12,13,14,15]. Additional complement-independent mechanisms impairing the NMJ in AChR-MG include blockage of the AChR binding site and AChR modulation, i.e., endocytosis and degradation of cross-linked AChR molecules by autoAbs [26]. At present, indicators of that might be the prevailing mechanisms at the individual level are lacking or ‘difficult to investigate’, and biomarkers of complement activation in AChR-MG patients have not been extensively studied. Their identification is now becoming a medical need, due to the demonstrated therapeutic efficacy of anti-complement biological drugs (i.e., eculizumab, zilucoplan) in gMG patients [19,20,21,22,23]. Heterogeneity of anti-AChR Ab-mediated complement activity was very recently described in AChR-MG patients by Obaid and colleagues, who developed an assay based on EK293T cell line-modified using CRISPR/Cas9 genome editing to disrupt complement regulator gene expression, for measuring autoAb-mediated MAC formation by flow cytometry [31]. By means of this system, a subset of AChR-MG patients was found to have high complement activity, likely due to complement activation as a major disease mechanism, whereas the remaining patients showed low activity, likely because they harbored autoAbs-mediating pathology via complement-independent mechanisms [31]. These data highlight that biomarkers of complement activation as an Ab-mediated pathogenic mechanism in individual AChR-MG patients could anticipate a successful response to anti-complement therapies, allowing early selection of patients who could benefit from complement inhibition. Tailoring the treatment might be crucial for the future positioning of anti-complement therapies in the MG treatment algorithm, overcoming the issues related to the high costs of these innovative biological drugs, and to the inter-individual variation in the improvement degree registered in the clinical studies [20,23,32]. The identification of easy-to-measure complement activation biomarkers, such as plasma complement system proteins, could promote the introduction of complement-related biomarkers into the MG clinical practice.

By performing a comprehensive plasma complement protein analysis, we showed significantly reduced levels of both C2 and C5 proteins in plasma of AChR-MG patients compared to controls, indicative of ongoing complement activation and consumption of these components (Figure 1). This reduction was not observed in MuSK-MG patients versus controls. Due to the important role of the classical complement pathway in AChR-MG [4,5], and to unchanged plasma levels of the alternative pathway proteins Factor B and properdin [30], we suggest that the observed C2 and C5 consumption is mainly due to classical complement pathway activation in AChR-MG patients. Complement system activation in plasma is a feature of a large number of diseases, in which consumption of complement components and an increase in activation products occurs [33]. C5 is responsible for the final step of complement cascade through its cleavage in the anaphylatoxin C5a, a potent mediator of inflammation [34], and C5b, which recruits C6, C7, C8, and C9 to form MAC [6]. In line with C5 consumption, we observed that C5a levels were significantly increased in the group of AChR-MG, but not in MuSK-MG, patients versus controls (Figure 2), reflecting complement activation. Contrariwise to C2 and C5, a significant increase in C3 plasma levels was observed in AChR-MG patients (Figure 1), which could be related to innate immune response and inflammation. Indeed, C3 plays a pivotal role in innate immune activation [27], which has been widely associated with AChR-MG pathogenesis [35]. Based on the evidence of extra-hepatic C3 production by T cells upon activation, the C3 increase in AChR-MG could also be related to T cell activation [36]. C3 is the central and most abundant complement component, acting as a substrate of C3 convertases, which cleaves C3 into the anaphylatoxin C3a and the larger C3b fragment [33]. Of note, the C3 increase in AChR-MG patients was associated with a significant increase in C3b (Figure 2), and the C3 levels were positively correlated with those of C3b in these patients (Supplementary Figure S1B). Since C3b forms the C5 convertase C4b2a3b, C3b increase was also in line with reduced C5 and increased C5a levels in AChR-MG patients (Figure 1 and Figure 2). The end-product of the complement cascade is the terminal complement complex C5b-9, which may remain in the plasma as soluble C5b-9 or be inserted in the cell membrane as MAC [33]. We did not detect significant changes in plasma C5b-9 levels in our cohort of AChR-MG patients compared to controls (Figure 2), suggesting that complement activation and MAC formation at the NMJ do not lead to a detectable increase in soluble C5b-9 in plasma.

Plasma levels of the altered complement proteins (C2, C3, C3b, C5, and C5a) in the AChR-MG group did not linearly correlate with disease severity evaluated by MGC and MG-ADL scores, nor with anti-AChR Ab titers. This could be due to: (i) the reduced sample size, with individual variation in the complement protein levels, implying the importance of further studies on larger patients’ cohort; (ii) the contribution of additional (e.g., genetic, molecular, immunological) factors in determining the complexity of AChR-MG clinical status/severity; and (iii) other complement-independent autoAb-mediated mechanisms impairing the NMJ, and hence determining disease severity, in some AChR-MG patients, as also postulated by Obaid and colleagues [31]. Nevertheless, the identified profile could represent a biomarker of complement activation to guide the choice of anti-complement therapies, among other treatment options, such as treatment with other not complement-related biological drugs in patients resistant or intolerant to immunosuppressive drugs. We did not find significant differences between pharmacological remission and symptomatic patients, according with a lack of correlation between complement protein levels and disease severity. A recent study by Keller and colleagues [37] showed increased serum concentration of activated complement proteins, such as C5a and C3a, in patients with myelin oligodendrocyte glycoprotein (MOG)-antibody (Ab)-associated diseases (MOGAD), suggesting that complement proteins may contribute to central nervous system tissue damage, and may be therapeutic targets in these conditions. In line with our data, the Authors did not find differences in the systemic complement activation profile between MOGAD patients classified as clinically active and patients in remission [37], which could be explained by the contribution of additional factors, along with complement system, to the complex mechanisms leading to the disease. 

No difference was previously found in C3, C4, and C5a levels between AChR-MG patients under symptomatic and immunosuppressive therapies [38]. Accordingly, we found comparable complement protein levels between patients treated and untreated with corticosteroids, and no correlation between these levels and the duration of IS treatment, suggesting that the observed complement activation profile was not related to the treatment. Indeed, it is unlikely that immunosuppression may increase complement activation, although, the limited number of corticosteroid-naïve versus corticosteroid-treated patients does not allow a definite conclusion. MBL showed a trend to be reduced in corticosteroid-treated compared to untreated AChR-MG patients, suggesting a possible effect of immunosuppressive therapy on its plasma levels that should be further explored.

Despite the clear involvement of complement in MG, studies assessing complement parameters as possible immunological biomarkers for AChR-MG are limited: (i) Romi and colleagues found a reduction in C3 and C4 serum levels in AChR-MG patients with high Ab titers, suggesting an increased in vivo complement consumption unrelated to clinical severity [9]; (ii) Liu and colleagues showed lower C3 levels in serum of AChR-positive compared to AChR-negative gMG patients and healthy controls [39], suggesting complement activation-dependent C3 consumption; (iii) Aguirre and colleagues assessed serum C3 and C4, and plasma C5a in AChR-MG patients, and showed a positive correlation of C5a levels with MG severity according to MG-ADL, but these levels were not different from those of healthy controls [38]. The discrepancy among data from different studies is likely due to technical issues related to the different methods used for complement protein quantification, and particularly to sample handling and storage and sample types (serum versus plasma) used in the analysis. Indeed, complement activation measurement requires that practical guidelines to avoid biased results, due to in vitro complement activation and consumption, are rigorously followed [33,40]. Properly collected EDTA-plasma is suitable for analysis of individual components and activation products, due to the EDTA inhibitory effect on complement activation, since EDTA chelates Ca^2+^ and Mg^2+^, thereby blocking the function of the C1 complex and the two C3 convertases. On the contrary, if serum is prepared, further activation of the sample in vitro is possible and it can be used for functional testing (e.g., hemolytic assays) [33,40]. Recommendations for complement laboratory analyses prompted us to collect MG patients’ and controls’ plasma samples ad hoc for this study to obtain confident results.

## 4. Materials and Methods

### 4.1. Patients and Samples

Twenty-three MG patients, including eighteen AChR-positive and five MuSK-positive MG patients, and fourteen age- and sex-matched healthy controls without autoimmune diseases or infections at the time of blood collection, were included in our study (Table 1). Disease severity in MG patients was evaluated at blood collection by means of the MG-ADL scale [41] and MGC score [42].

Plasma samples were collected from patients and controls following guidelines for laboratory assessment of complement activation [40]. EDTA-containing tubes were used for blood collection and immediately placed on ice before centrifugation; aliquots of the isolated plasma were immediately transferred at −80 °C. Each plasma aliquot was frozen and thawed only once, and processed immediately after thawing on ice for complement protein assay.

The study was approved by the Fondazione IRCCS Istituto Neurologico Carlo Besta Ethics Committee (No. 586/2014). Patients and controls signed an informed consent form for using their biological samples for research.

### 4.2. Multiplex Immunoassays for Complement Panel Analysis

The human complement Panel 1 and Panel 2 bead-based multiplex assay kits (Millipore Burlington, MA, USA) were used for simultaneous quantification of complement components and activation products of the classical, lectin, and alternative pathways in plasma samples. Complement Panel 1 is a seven-plex kit allowing measurement of C2, C4b, C5, C5a, Factor D, MBL, and Factor I. Complement Panel 2 is a six-plex kit allowing measurement of C1q, C3, C3b, C4, Factor B, and Factor H. The assays were performed according to the manufacturer’s guidelines and the plates were read on the BioPlex 200 system (Bio-Rad Laboratory, Hercules, CA, USA), powered by the Luminex xMAP technology. Plasma dilution (1:200 for Panel 1, and 1:40,000 for Panel 2) was optimal for simultaneous assessment of all complement proteins, except C2, Factor D, Factor I, and C5a, because protein levels were out of the standard curve range. C2 and C5a were assessed separately by Enzyme-linked immunosorbent assays (ELISA), as described below.

### 4.3. ELISA

The Human Complement Component C2 (Novus Biologicals, Littleton, CO, USA), Human C5a (Invitrogen Corporation, Waltham, MA, USA), Human C5b-9 (BD Biosciences, San Jose, CA, USA), and Human Properdin (Invitrogen, Carlsbad, CA, USA) ELISA kits were used to measure plasma concentration of C2, C5a, C5b-9, and Properdin, respectively. We used assay protocol and sample dilution in accordance with the manufacturers’ instructions. C5b-9 assay was optimized using a 100-fold sample dilution.

### 4.4. Statistical Analyses

Non-parametric distributed data were analyzed by the Kruskal–Wallis test with Dunns post hoc test for multiple comparisons, or by the Mann–Whitney test for comparison of two groups. The Grubbs’ test was used to identify and exclude significant outliers (corresponding to values with 2.5 standard deviations from the mean) in our datasets. Correlation analyses were performed using the Spearman rank correlation coefficient. Receiver operating characteristic (ROC) curves were applied to estimate the biomarker potential of plasma complement proteins for discriminating between AChR-MG patients and healthy controls. *p* values < 0.05 were considered significant. GraphPad Prism v8.0 (La Jolla, San Diego, CA, USA) was used for data analyses.

## 5. Conclusions

Our overall data revealed a plasma complement profile associated with AChR-MG, consisting of a significant C2 and C5 reduction, and increased C3, C3b, and C5a levels (Table 2). This profile is compatible with complement activation and fits with the immune pathogenesis of MG, but we need to verify its reliability as a biomarker in a larger patients’ cohort that will enable a cross-check of its use in a stratified population. Further studies on larger series of patients stratified according to disease clinical variables, e.g., clinical severity or status and treatment, should be performed to find the correlation between complement activation status and degree of clinical impairment, to help the clinician in selecting patients as candidates for complement inhibition.

## Figures and Tables

**Table 1:** Summary of the Main Features of AChR-MG and MuSK-MG Patients, and Healthy Controls Included in the Study.

	Healthy Controls (*n* = 14)	AChR-MG Patients (*n* = 18)	MuSK-MG Patients (*n* = 5)
Sex (F:M)	9:5	10:8	5:0
Age at onset (years, mean ± SD)	-	47.9 ± 16.7 ^1^	39.0 ± 5.5 ^2^
Age at blood collection (years, mean ± SD)	33.4 ± 8.7	54.0 ± 13.2	54.8 ± 14.4
Disease duration (years, mean ± SD)	-	6.8 ± 5.5 ^1^	13.0 ± 11.2 ^2^
MGC at blood collection (mean ± SD)	-	7.8 ± 9.5 ^3^	2.4 ± 2.2
MG-ADL at blood collection (mean ± SD)	-	5.3 ± 5.6 ^3^	2.2 ± 1.8
Immunosuppressive drugs	-	15	4
Thymectomy ^4^	-	7	1
Thymic histology (thymoma) ^5^	-	3	0

^1^ Information on age at onset and disease duration was not available in 3 of the 18 AChR-MG patients. ^2^ Information on age at onset and disease duration was not available in 1 of the 5 MuSK-MG patients. ^3^ MGC and MG-ADL were not available in 2 of the 18 AChR-MG patients. ^4^ Number of patients thymectomized at the time of blood collection. ^5^ Patients without thymoma had hyperplastic thymus.

**Table 2:** Complement Activation Profile in Plasma of AChR-MG Patients.

	AChR-MG Versus Healthy Controls
Complement components	
C2	Decreased **
C3	Increased **
C5	Decreased **
Complement activation products	
C3b	Increased **
C5a	Increased *

* *p* < 0.05; ** *p* < 0.01.

## Data Availability

The data presented in this study are available on request from the corresponding author, P.C.

## Supplementary Materials

The following supporting information can be downloaded at: https://www.mdpi.com/article/10.3390/biomedicines10061360/s1, Figure S1: Quantification of complement components (C2, C3, C5, and MBL) and activation products (C3b, C5a) in plasma of AChR-MG patients stratified as patients under pharmacological remission (PhR-MG) and with generalized symptoms (gMG), and healthy controls (HC). C3, C5, MBL, and C3b levels were obtained by multiplex immunoassays using Luminex technology; C2 and C5a levels were assessed by specific ELISA. For MBL data analysis, one outlier in the PhR-MG group was excluded based on the Grubbs’ test. No outliers were identified in the other protein datasets. (A) No difference in complement component C2, C3, C5, and MBL concentration values was found between PhR-MG and gMG patients’ subgroups. C2, C5, and MBL concentrations were lower in both PhR-MG and gMG subgroups compared to controls, although, significant results were reached only for C2 and C5 levels in PhR-MG patients. C3 levels were higher in both PhR-MG and gMG subgroups compared to controls. (B) Positive correlation estimated by Spearman’s correlation analysis between C3 and C3b levels in plasma of AChR-MG patients. (C) No difference in the levels of complement activation products C3b and C5a was found between PhR-MG and gMG patients’ subgroups. C3b levels were significantly higher in both PhR-MG and gMG subgroups compared to controls. C5a levels showed a trend to be increased in PhR-MG and gMG patients compared to controls, but results were not significant.
