# Supplementary material for: Complement Activation Profile in Myasthenia Gravis Patients: Perspectives for Tailoring Anti-Complement Therapy
Source: Biomedicines. 2022 Jun 9;10(6):1360. doi: 10.3390/biomedicines10061360 (PMC9220000; doi:10.3390/biomedicines10061360)
Supplement: Supplementary file 1 [file biomedicines-10-01360-s001.zip › biomedicines-1696478-supplementary.pdf]

Supplementary Data

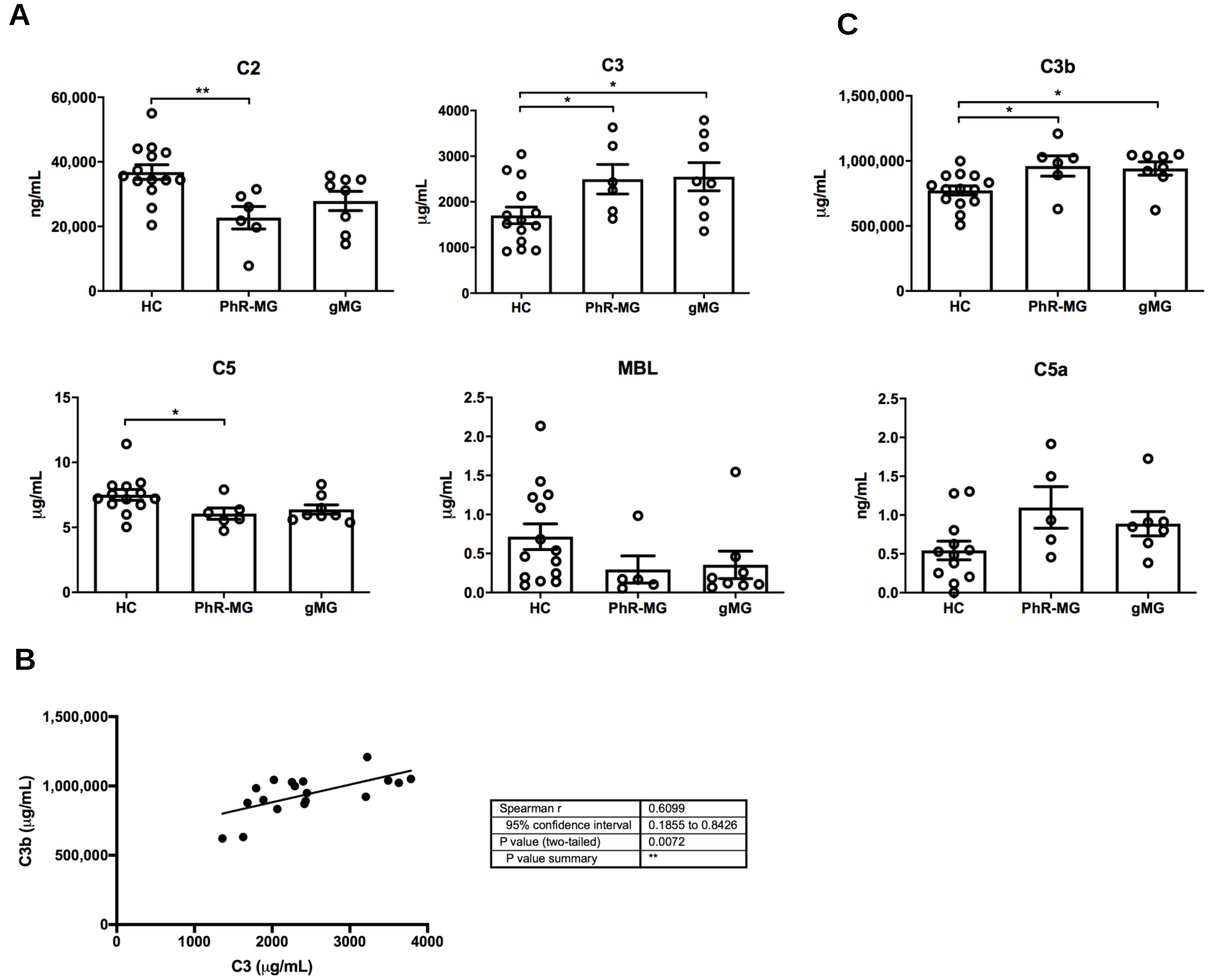

Spearman r	0.6099
95% confidence interval	0.1855 to 0.8426
P value (two-tailed)	0.0072
P value summary	**

Supplementary Figure S1. Quantification of complement component (C2, C3, C5, MBL) and activation products (C3b, C5a) in plasma of AChR-MG patients stratified as patients under pharmacological remission (PhR-MG) and with generalized symptoms (gMG), and healthy controls (HC). C3, C5, MBL and C3b levels were obtained by multiplex immunoassays using Luminex technology; C2 and C5a levels were assessed by specific ELISA. (A) No difference in complement component C2, C3, C5 and MBL concentration values was found between PhR-MG and gMG patients' subgroups. C2, C5 and MBL concentration was lower in both PhR-MG and gMG subgroups compared to controls, although significant results were reached only for C2 and C5 levels in PhR-MG patients. C3 levels were higher in both PhR-MG and gMG subgroups compared to controls. (B) Positive correlation estimated by Spearman's correlation analysis between C3 and C3b levels in plasma of AChR-MG patients. (C) No difference in the levels of complement activation products C3b and C5a was found between PhR-MG and gMG patients' subgroups. C3b levels were significantly higher in both PhR-MG and gMG subgroups compared to controls. C5a levels showed a trend to be increased in PhR-MG and gMG patients compared to controls, but results were not significant.
